# Supplementary material for: Spatial and temporal scales of variability for indoor air constituents
Source: Commun Chem. 2021 Aug 12;4:110. doi: 10.1038/s42004-021-00548-5 (PMC9814873; doi:10.1038/s42004-021-00548-5)
Supplement: Supplementary file 1 — Supplementary information. [file 42004_2021_548_MOESM1_ESM.pdf]

Supplementary Methods.

Kinetic Model Description

A schematic of the model used in this work is shown in Fig. 1a and includes a gas-phase, boundary layer, and an aqueous bleach layer. The model has previously been applied to bleaching experiments during HOMEChem and is described in detail in that publication [1]. Processes included in the model are air exchange, uptake to particulate matter and indoor surfaces, photolysis, gas-phase reactions and reactions in the aqueous bleach. Transport of semi-volatile species between the gas-phase and the bleach requires transport through a boundary layer and is treated using our previously developed kinetic multi-layer model of the boundary layer (KM-BL)[2]. An assumption made in the kinetic model is that the indoor gas-phase is well-mixed. The diffusion coefficient of species ($D_{h,Z}$) at a height (h) above the bleach is dependent on its diffusion coefficient under non-turbulent conditions ($D_{g,Z}$) and on the turbulence intensity (K_e) as follows[3,4]:

$$D_{h,Z} = D_{g,Z} + K_e h^2 \quad \text{(E1)}$$

Reversible adsorption to the surface of the bleach and partitioning into the bleach were also treated in the model. Note that observations of chlorinated and nitrogenated compounds could not be reproduced by the model without multiphase chemical processes, as demonstrated in our previous study[1]. A full list of reactions, rate coefficients, diffusion coefficients and partitioning coefficients used in the model are summarized in a previous publication [1].

The kinetic model provided inputs to the Computational Fluid Dynamics (CFD) model including the concentrations of HOCl, $ClNO_2$, chloramines, and NH_3 directly above the bleach surface at different times. The most important gas-phase reactions and uptake coefficients which were responsible for controlling the concentrations of species of interest were identified using the kinetic model and sensitivity tests (Supplementary Table 1). These reactions and uptake coefficients are included in the CFD simulations. Uptake coefficients to room surfaces were calculated assuming a total room surface area of 430 m^2. In addition to the reactions and uptake coefficients included in Supplementary Table 1 an indoor NH_3 production rate of 1.24×10^8 cm^{-3} s^{-1} was included in both the kinetic model and CFD simulations. A previous study has suggested primary emissions of Cl_2 and $ClNO_2$ from the bleach due to solution impurities.[5] This possibility was not considered in the model due to a lack of experimental constraints,[6] which may be one of the reasons for the difference between measurements and modeling (Supplementary Figure 2).

Computational Fluid Dynamics simulations

Spatial distributions of gas-phase bleach products and subsequent chemical reaction products were simulated under various indoor environmental conditions using a CFD model. The CFD model geometry was designed by mimicking air flow and emission conditions of the bleach products observed in the measurement campaign (Supplementary Figure 1)[7]. The simulation domain involved the total house air volume of 250 m^3 along with the floor area of 111 m^2 and entire indoor surface area of 420 m^2. In the model, the bleach solution was applied in the cleaning area of the living room (highlighted with the blue marks in Supplementary Figure 1) with the total area of 40 m^2. The model also simulated outdoor air infiltration into the house at a rate of 0.7 h^{-1} and indoor air recirculation through the central air handling unit at an air mixing rate of 8 h^{-1}, as characterized during the HOMEChem campaign[7]. The average room air temperature was 25 °C.

The model also simulated solar radiation through windows (three yellow triangular columns in Supplementary Figure 1). The areas are 2, 4, and 2 m^2 for the three windows, respectively, and the solar zenith angle was 60 degrees. The photolysis rate coefficient was considered uniform in the direct solar radiation zone while in the diffuse sunlight zone it was calculated as a 2% value of the direct sunlight zone, considering diffusions and reflections of photons in the non-sunlit zone.

A total of eleven chemical reaction equations were simulated (See Supplementary Table 1). Four species, HOCl, $ClNO_2$, NCl_3, and $ClNO_2$, were generated directly from the cleaning surface. The concentrations directly above the bleach surface calculated from the kinetic model were inputted in the CFD model. In reaction 1 (R1), HOCl reacts with chlorine ions (Cl^-) on aerosol surfaces in the ambient air of the room, producing chlorine (Cl_2) and water vapor. It was assumed that aerosol particles, where HOCl uptake occurred on the surface, were distributed uniformly throughout the room, while particles were not explicitly resolved in CFD. Note that direct solar radiation in the sunlit zone photolyzed Cl_2, $ClNO_2$ and HOCl, thereby generating Cl and OH radicals (see reactions R2 - R4). OH production rates in the dark zone and the sunlit zone were calculated by the INDCM (INdoor Detailed Chemical Model) and set in the CFD model (R5). OH radicals were consumed by the fast reactions with HOCl (R6) and indoor VOCs with a pseudo-first order reaction rate of 65 s^{-1} (R7). Based on the experimental conditions (*4*), NH_3 reacts on the cleaning surface (R8) and the modeled background mixing ratio was 26 ppb.

The CFD model simulates surface uptake fluxes using the following equation:

$$F_i = \frac{\gamma_i \omega_i}{4} C_i \qquad \text{(E2)}$$

where F_i is uptake flux of species, γ_i is uptake coefficient (or reaction probability), ω_i is thermal velocity, and C_i is the gas-phase concentration of species i adjacent to the wall. NH_3 is lost due to uptake to the bleach surface (R8). Cl_2, $ClNO_2$, and NCl_3 are deposited to all indoor surfaces such as the walls, floor, and ceiling (R9 - R11).

To simulate the turbulent indoor air flow associated with the supply air jets and interior surfaces, the Menter k-ω shear stress transport turbulence model was utilized, where two turbulence variables, kinetic energy (k) and specific dissipation (ω) described turbulent eddy scales and kinetic energy. The model results were validated based on the mass and energy balance equations for gas-phase species chemical reactions as described in previous CFD studies[8,9]. In addition, the CFD model results were validated further by comparing time-varying concentrations of OH, HOCl, NCl_3, and NH_3 observed in the measurement campaign (Fig. 1) [1]. When considering the error (10 %) of the measurements, the CFD model agrees well with the general pattern of the experimental conditions. As OH was measured only at P7, it is challenging to directly validate horizontal and vertical distributions. However, when P7 was under dark conditions during other bleach cleaning events, OH concentrations were near the detection limit of the instrument (approximately 1×10^6 cm^{-3}). Elevated concentrations of OH during bleach cleaning events were only observed when P7 was illuminated. These observations serve as an indirect validation of strong concentration gradients between dark and sunlit zones.

Gas-phase chemistry modeling

Modeling of OH concentrations was carried out using the INDCM (INdoor Detailed Chemical Model)[10], a near explicit photochemical box model constructed based on a comprehensive chemical mechanism (the Master Chemical Mechanism, MCM v3.3.1, http://mcm.leeds.ac.uk/MCM/) [11]. The MCM represents the gas-phase degradation of ~143 VOCs, with each undergoing reaction with NO_3 and OH radicals, O_3, and photolysis where relevant [11]. The INDCM also includes terms that consider exchange with outdoors, internal emissions, photolysis, and deposition to surfaces as described in detail previously [10,12]. For this work, the concentrations of NO_x, O_3, 48 VOCs, 3 inorganic Cl-containing species, 9 photolysis coefficients, outdoor light attenuation factors, H_2O, temperature, and occupancy were inputted as constraints for the INDCM, based on observed values from the 8th June during the HOMEChem campaign.

Outdoor NO_x, O_3 and VOCs were also used to provide typical values where available. The model was then used to predict the radical concentrations to compare with measurements.

The OH reactivity is defined for a chemical species X as the product of the second-order rate coefficient for the reaction of X with OH radicals with the concentration of X (k^{II} [X]). The total OH reactivity k_{OH}, which is the inverse of the OH lifetime, is calculated by summing the OH reactivity for each chemical species X. The INDCM contains an explicit chemical mechanism that includes all of the important OH loss routes, and the reactivity is then driven by the measured concentrations used as inputs. As well as providing an estimate of OH reactivity, the INDCM was also used to provide OH production rates as inputs for the CFD model. The production rate was calculated every minute and a 10-min running average was applied for inputting into the CFD model as shown in Supplementary Table 1.

Spatial and temporal scales

Spatial and temporal scales of indoor species are determined by loss rates due to building ventilation, surface deposition, photolysis, and chemical reactions. Supplementary Table 2 summarizes the major processes considered in evaluating spatial and temporal scales of gas-phase species and particulate matters indoors. The overall first-order decay rate coefficient (s^{-1}) is the sum of coefficients of the air exchange rate (λ), deposition rate (d), coagulation rate (c), photolysis rate (j), and first-order chemical reaction rate (k^{I})[1,4,8,9,13-15]. The half-life ($t_{\frac{1}{2}}$) of species is calculated as follows:

$$t_{\frac{1}{2}} = \frac{\ln 2}{(\lambda + d + c + j + k^{I})} \quad \text{(E3)}$$

The spatial scale is the transport distance of a given species for the half-life. As the air exchange rate varied from 0.5 to 5 h^{-1}, the average air velocity magnitude ($\bar{v}$) increased from 0.03 $m\ s^{-1}$ to 0.05 $m\ s^{-1}$ [8,9]. The velocity magnitude of 0.03 $m\ s^{-1}$ was selected for the baseline condition. The spatial scale is calculated as follows:

$$\text{Distance} = \bar{v}\, t_{\frac{1}{2}}$$

Gas-phase species:

We calculated and summarized the spatial and temporal scales of selected indoor gas-phase species based on the present study of bleach cleaning and previous studies including ozone

interactions with the human surface *(8)* and photochemical reaction of HONO generated from a gas stove *(7)*. The background indoor concentrations/mixing ratios of gas-phase species were OH of 3×10^5 cm^{-3}, O_3 of 4 ppb, VOCs of 100 ppb, NO_2 of 5 ppb, and NO of 2 ppb. Reaction rate coefficients were based on the INDCM[10,12] and the MCM[11].

Lifetimes of radicals shown in Fig. 3 are not instantaneous lifetimes calculated with all possible loss reaction, but they represent steady-state lifetimes, which are calculated by considering the termination reactions of propagation reactions of radical families. The reactions of OH with VOCs do not terminate the radicals, but will propagate them to RO_2 and HO_2. RO_2 will quickly reform HO_2 (e.g., via $RO_2 + NO \rightarrow RO + NO_2$, $RO + O_2 \rightarrow RCHO + HO_2$) and then OH radicals can be regenerated via $HO_2 + NO \rightarrow OH + NO_2$ given typical indoor NO concentrations. Thus, OH, HO_2, and RO_2 radicals are tightly coupled and it is difficult to separate these three radical species with relatively high NOx concentrations indoors; hence, it is most reasonable to consider the lifetime of the chemical family RO_x (= $OH + HO_2 + RO_2$)[16]. Based on MCM and INDCM simulations, the chemical lifetime of RO_x is determined by the following radical termination reactions: $OH + NO \rightarrow HONO$, $OH + NO_2 \rightarrow HNO_3$, $RO_2 + NO \rightarrow RONO_2$ (organic nitrate), $RO_2 + NO_2 \rightarrow ROONO_2$ (peroxynitrate). Note that $RO_2 + NO$ can lead to other products and the branching ratio for the formation of $RONO_2$ is assumed to be 0.23, representing limonene, one of the most abundant VOCs indoors.

Cl radicals do not propagate in the same way, so the lifetime of Cl radical is controlled by the reaction with VOCs. Note that $Cl + O_3 \rightarrow ClO + O_2$ is not considered as a Cl loss reaction, as ClO can be quickly converted back to Cl by $ClO + NO \rightarrow Cl + NO_2$, which is substantially fast with a ppb level of NO indoors. NO_3 is lost via reactions with VOCs and the chemical lifetime is calculated with a 2nd order rate coefficient of 1.2×10^{-11} cm^{-3} s^{-1}, which is the rate coefficient for the reaction with limonene[17]. Chemical lifetimes of closed-shell species (e.g., non-radicals) are calculated based on their reactions with OH or O_3, as shown in Supplementary Table 2.

Particulate matter:

We report spatial and temporal scales of six different sizes of particles with particle diameters of 3 nm, 100nm, 1 μm, 5 μm, 10 μm, and 100 μm, as described in Supplementary Table 2. Nano-size particles decrease their concentrations by coagulation, deposition, and ventilation[13,14,18], while micro-size particles decay mostly by deposition and ventilation[3,4,15,19]. The

coagulation of nano-size particles contributes to 20% of the deposition rate, whereas it is negligible for micro-size particles[18]. Particle deposition to indoor sources is enhanced for nano-size particles (< 10 nm) due to Brownian and turbulent diffusion, while gravitational settling is dominant for larger particles (> 5 μm). These size-varying loss mechanisms determine the half-life and spatial transport scales.

Supplementary Table 1. List of chemical reactions considered in computational fluid dynamics simulations.

Reaction	Rate coefficient
1) HOCl +Aerosols → Cl_2	γ= 0.4
2) Cl_2 + hv → 2Cl	*J_{Cl2}= 2.6 × 10^{-4} s^{-1}
3) HOCl + hv → OH + Cl	*J_{HOCl}= 2.4 × 10^{-5} s^{-1}
4) $ClNO_2$ + hv → NO_2 + Cl	*J_{ClNO2}= 1.7 × 10^{-5} s^{-1}
5) OH production	P_{OH} = 10^7 cm^{-3} s^{-1} (dark zone)<br>P_{OH} in sunlit zone<br>0 - 10 min: 2.42×10^8 cm^{-3} s^{-1}<br>10 - 20 min: 1.55×10^9 cm^{-3} s^{-1}<br>20 - 30 min: 1.88×10^9 cm^{-3} s^{-1}<br>30 - 40 min: 6.18×10^8 cm^{-3} s^{-1}<br>40 - 50 min: 1.82×10^8 cm^{-3} s^{-1}
6) HOCl + OH → Products	k_6= 5.0 × 10^{-13} cm^3s^{-1}
7) OH → Loss	k_7 = 65 s^{-1}
8) NH_3 + Bleach surfaces → Products	γ= 1.9 × 10^{-2}
9) Cl_2 + Room surfaces → Products	γ= 3.1 × 10^{-5}
10) $ClNO_2$ + Room surfaces → Products	γ= 9.2 × 10^{-6}
11) NCl_3 + Room surfaces → Products	γ= 8.4 × 10^{-6}

γ is uptake coefficient. * Photolysis rate constant (J) values are the value in the direct solar radiation zone near windows. 2% of these values are set in the rest of the room.

Supplementary Table 2. Temporal and spatial scales of selected indoor species and particulate matter. Reaction rate coefficients (k^{II}) were based on the INDCM[10,12] and the MCM[11]. Photolysis rates, gas-phase deposition rates and particulate matter coagulation and deposition rates are based on literature values. [1,4,8-15,18,20-22]

Species	k^{II} ($cm^3\ s^{-1}$)	Chemical# reaction (k^I) (s^{-1})	Photolysis (j) (s^{-1})	Deposition (d) (s^{-1})	Sum* (s^{-1})	Half-life (s)	Distance (m)
Gas-phase species							
RO_x	1.2×10^{-11} ($OH+NO_2$)<br>1.0×10^{-11} (OH+NO)<br>9.0×10^{-12} (RO_2+NO)<br>9.0×10^{-12} (RO_2+NO_2)	3.2			3.2	0.22	6.6×10^{-3}
NO_3	1.2×10^{-11} (VOC)	0.59	10^{-3}		0.59	1.2	3.5×10^{-2}
O_3	7.3×10^{-14} (OH)<br>1.0×10^{-15}(VOC)	4.9×10^{-5}	4.3×10^{-6}	1.3×10^{-3}	1.5×10^{-3}	4.7×10^{2}	14
H_2O_2	1.7×10^{-12} (OH)	1.2×10^{-6}	4.5×10^{-9}	1.4×10^{-3}	1.5×10^{-3}	4.7×10^{2}	14
HONO	6.1×10^{-12} (OH)	4.3×10^{-6}	1.4×10^{-6}	1.2×10^{-3}	1.3×10^{-3}	5.2×10^{2}	15
HCl	7.8×10^{-13} (OH)	5.5×10^{-7}		5.3×10^{-3}	5.4×10^{-3}	1.3×10^{2}	3.8
Cl	8.5×10^{-11} (VOC)	210			210	3.3×10^{-3}	9.9×10^{-5}
Cl_2	5.9×10^{-14} (OH)	4.2×10^{-8}	2.7×10^{-6}	4.0×10^{-3}	4.2×10^{-3}	1.7×10^{2}	5.0
HOCl	5.0×10^{-13} (OH)	3.5×10^{-7}	1.9×10^{-7}	1.3×10^{-3}	1.4×10^{-3}	4.9×10^{2}	15
$ClNO_2$	-	0	2.5×10^{-7}	1.1×10^{-3}	1.2×10^{-3}	5.5×10^{2}	17
HCHO	9.0×10^{-12} (OH)	6.3×10^{-6}	2.1×10^{-8}	1.1×10^{-3}	1.2×10^{-3}	5.8×10^{2}	17
NO	1.0×10^{-11} (OH)<br>1.9×10^{-14} (O_3)	1.9×10^{-3}		4.2×10^{-4}	2.4×10^{-3}	2.8×10^{2}	9
NO_2	1.0×10^{-11} (OH)<br>3.2×10^{-17} (O_3)	1.0×10^{-5}	1.1×10^{-5}	4.2×10^{-4}	5.8×10^{-4}	1.2×10^{3}	36
CO_2	-	0			1.4×10^{-4}	5.0×10^{3}	150
6-MHO	4.3×10^{-16} (O_3)	4.2×10^{-5}		8.3×10^{-4}	1.0×10^{-3}	6.8×10^{2}	20
Isoprene	1.0×10^{-10} (OH)<br>1.3×10^{-17} (O_3)	7.2×10^{-5}		8.3×10^{-4}	1.0×10^{-3}	6.6×10^{2}	20
NH_3	-	0		1.3×10^{-2}	1.3×10^{-2}	52	1.5
Particulate matter			Coagulation (c) (s^{-1})	Deposition (d) (s^{-1})	Sum** (s^{-1})	Half-life (s)	Distance (m)
3 nm			1.9×10^{-3}	7.5×10^{-3}	9.6×10^{-3}	72.3	2.2
10 nm			1.9×10^{-3}	7.8×10^{-3}	1.1×10^{-3}	624	18.7
1 µm				5.7×10^{-5}	1.9×10^{-4}	3560	107
5 µm				9.7×10^{-4}	1.1×10^{-3}	624	18.7
10 µm				2.5×10^{-3}	2.6×10^{-3}	263	7.9
100 µm				0.12	0.12	5.8	0.2

$k^I = \Sigma\ k^{II}$ [X] (X= OH, O_3, NOx or VOC; [X] in the unit of molecules cm^{-3} at 1 atm, 298 K)

* Sum = λ (air exchange rate of 0.5 h^{-1} = $1.4\times10^{-4}\ s^{-1}$) + $k^I + j + d$

** Sum = $\lambda + c + d$

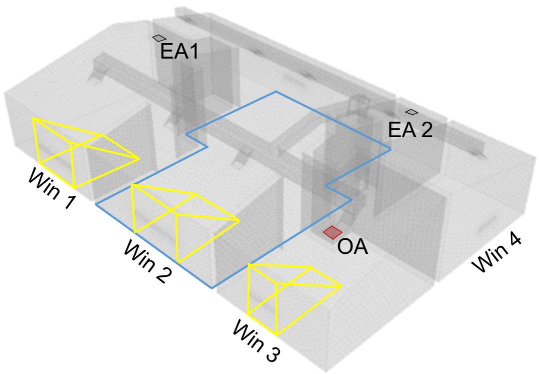

Supplementary Figure 1. Isometric view of computational fluid dynamics modeling geometry (Win: window, EA: exhaust air, OA: outside air). The yellow marks are solar radiation zones and the blue mark is the cleaning area.

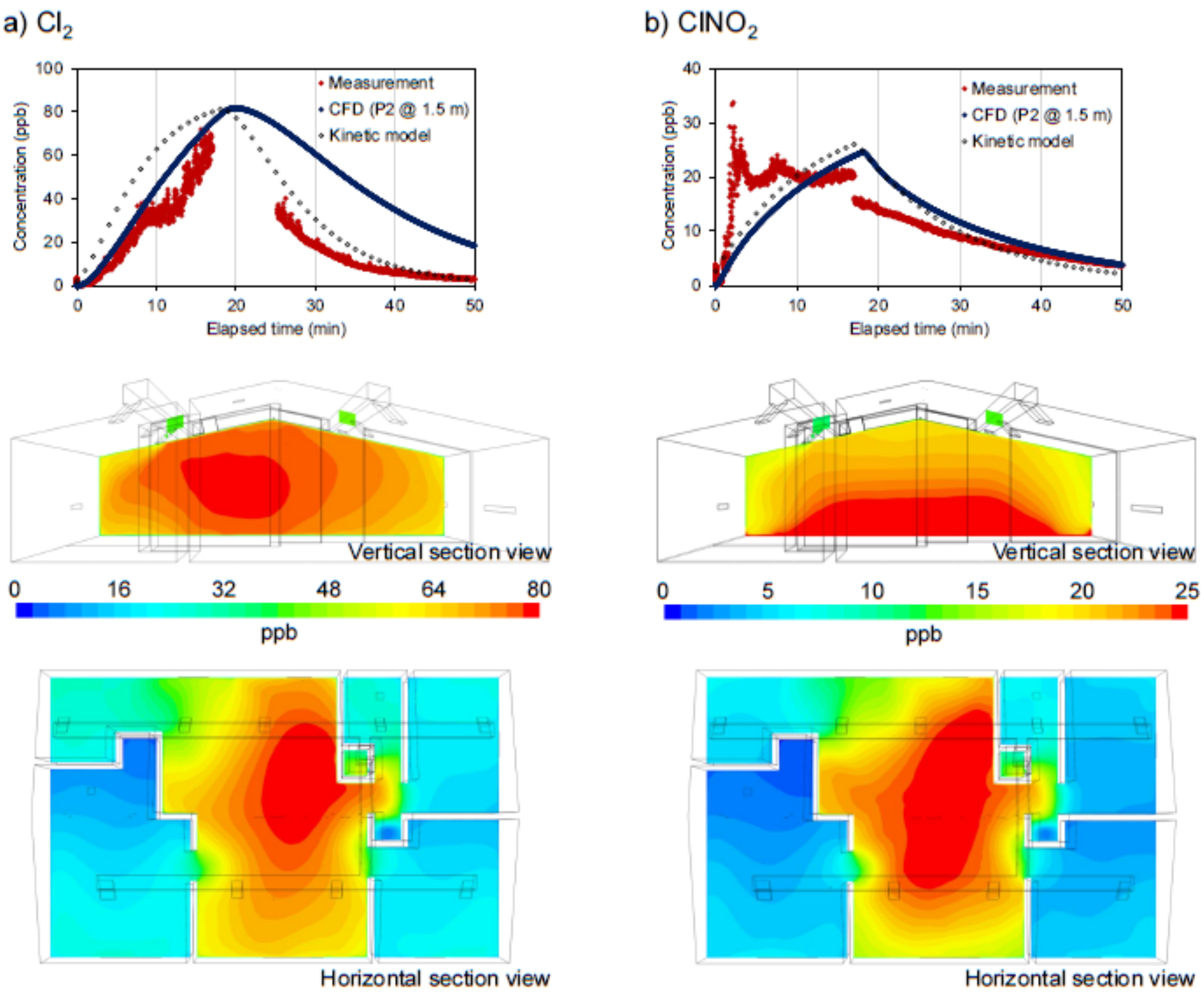

Supplementary Figure 2. Temporal evolution of (a) Cl_2 and (b) $ClNO_2$ as measured (red markers) and simulated by the CFD (solid lines) and the multiphase kinetic model (dark blue markers). Vertical and horizontal (1.5 m above the floor) spatial distributions at 18 minutes after the beginning of the cleaning.

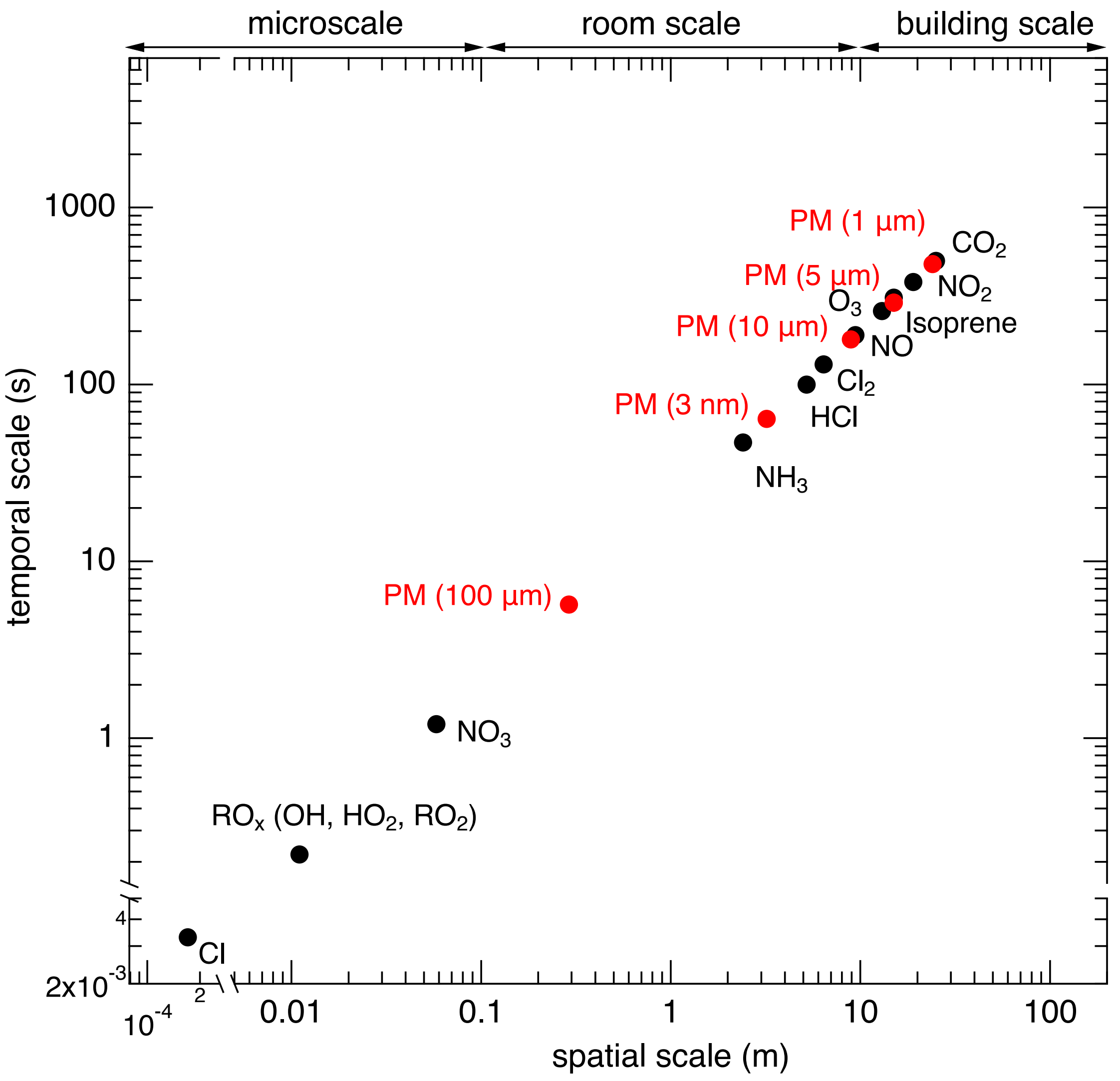

Supplementary Figure 3. Spatial and temporal scales of gas-phase species from chemical reactions indoors with an air exchange rate of 5 h^{-1}.

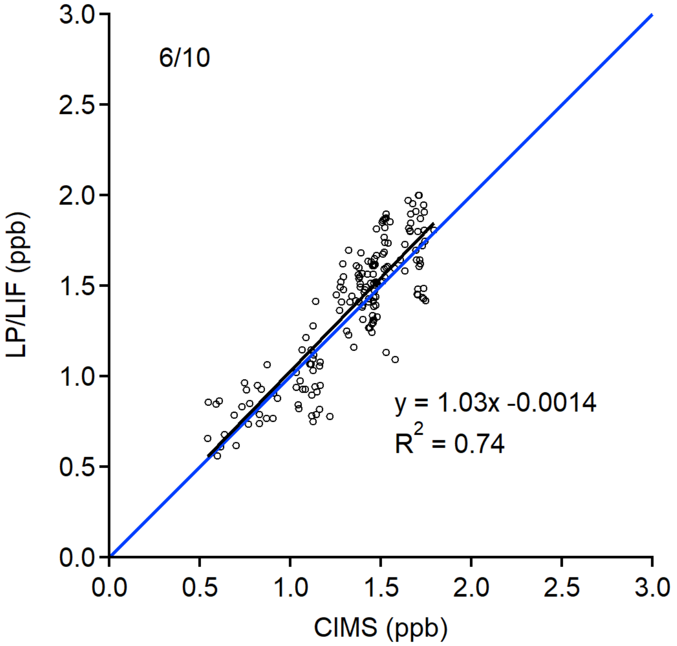

Supplementary Figure 4: Correlation plot of HONO measurements by the Laser-Photofragmentation LIF-FAGE instrument (LP-LIF) located at P7, and the Chemical Ionization Mass Spectroscopy instrument (CIMS) located at P2 during a HOMEChem sequential bleach mopping experiment.[6] The black line is a standard unweighted regression line and the blue line represents the 1:1 line.

Supplementary References.

1 Mattila, J. M. *et al.* Multiphase chemistry controls inorganic chlorinated and nitrogenated compounds in indoor air during bleach cleaning. *Environ. Sci. Tech.* (2020).

2 Morrison, G., Lakey, P. S., Abbatt, J. & Shiraiwa, M. Indoor boundary layer chemistry modeling. *Indoor Air* **29**, 956-967 (2019).

3 Corner, J. & Pendlebury, E. D. The coagulation and deposition of a stirred aerosol. *Proceedings of the Physical Society. Section B* **64**, 645 (1951).

4 Lai, A. C. K. & Nazaroff, W. W. Modeling indoor particle deposition from turbulent flow onto smooth surfaces. *J. Aerosol Sci.* **31**, 463-476 (2000).

5 Wong, J. P. S., Carslaw, N., Zhao, R., Zhou, S. & Abbatt, J. P. D. Observations and impacts of bleach washing on indoor chlorine chemistry. *Indoor Air* **27**, 1082-1090 (2017).

6 Mattila, J. M. *et al.* Multiphase Chemistry Controls Inorganic Chlorinated and Nitrogenated Compounds in Indoor Air during Bleach Cleaning. *Environ. Sci. Technol.* **54**, 1730-1739, doi:10.1021/acs.est.9b05767 (2020).

7 Farmer, D. K. *et al.* Overview of HOMEChem: House observations of microbial and environmental chemistry. *Environ. Sci. Processes Impacts* **21**, 1280-1300 (2019).

8 Won, Y., Waring, M. S. & Rim, D. Understanding the spatial heterogeneity of indoor OH due to photolysis of HONO using Computational Fluid Dynamics (CFD) simulation. *Environ. Sci. Technol.* **53**, 14470-14478 (2019).

9 Won, Y., Lakey, P. S. J., Morrison, G., Shiraiwa, M. & Rim, D. Spatial Distributions of Ozonolysis Products From Human Surfaces In Ventilated Rooms. *Indoor Air* **30**, 1229–1240, doi:10.1111/ina.12700 (2020).

10 Carslaw, N. A new detailed chemical model for indoor air pollution. *Atmos. Environ.* **41**, 1164-1179, doi:10.1016/j.atmosenv.2006.09.038 (2007).

11 Jenkin, M. E., Saunders, S. M. & Pilling, M. J. The tropospheric degradation of volatile organic compounds: a protocol for mechanism development. *Atmos. Environ.* **31**, 81-104, doi:http://dx.doi.org/10.1016/S1352-2310(96)00105-7 (1997).

12 Carslaw, N., Mota, T., Jenkin, M. E., Barley, M. H. & McFiggans, G. A Significant Role for Nitrate and Peroxide Groups on Indoor Secondary Organic Aerosol. *Environ. Sci. Technol.* **46**, 9290-9298, doi:10.1021/es301350x (2012).

13 Wallace, L. A., Emmerich, S. J. & Howard-Reed, C. Effect of central fans and in-duct filters on deposition rates of ultrafine and fine particles in an occupied townhouse. *Atmos. Environ.* **38**, 405-413 (2004).

14 Rim, D., Wallace, L. & Persily, A. Infiltration of outdoor ultrafine particles into a test house. *Environ. Sci. Technol.* **44**, 5908-5913 (2010).

15 Lai, A. C. K. & Nazaroff, W. W. Supermicron particle deposition from turbulent chamber flow onto smooth and rough vertical surfaces. *Atmos. Environ.* **39**, 4893-4900 (2005).

16 Whalley, L. K. *et al.* Evaluating the sensitivity of radical chemistry and ozone formation to ambient VOCs and NOx in Beijing. *Atmos. Chem. Phys.* **21**, 2125-2147, doi:10.5194/acp-21-2125-2021 (2021).

17 Ng, N. L. *et al.* Nitrate radicals and biogenic volatile organic compounds: oxidation, mechanisms, and organic aerosol. *Atmos. Chem. Phys.* **17**, 2103-2162, doi:10.5194/acp-17-2103-2017 (2017).

18 Wallace, L., Jeong, S. G. & Rim, D. Dynamic behavior of indoor ultrafine particles (2.3-64 nm) due to burning candles in a residence. *Indoor Air* **29**, 1018-1027 (2019).

19 Tellier, R., Li, Y., Cowling, B. J. & Tang, J. W. Recognition of aerosol transmission of infectious agents: a commentary. *BMC infectious diseases* **19**, 101 (2019).

20 Beneš, M. & Holub, R. F. Aerosol wall deposition in enclosures investigated by means of a stagnant layer. *Environ. Int.* **22**, 883-889 (1996).

21 Zhao, B. & Wu, J. Modeling particle deposition from fully developed turbulent flow in ventilation duct. *Atmos. Environ.* **40**, 457-466 (2006).

22 Cano-Ruiz, J., Kong, D., Balas, R. & Nazaroff, W. Removal of reactive gases at indoor surfaces: combining mass transport and surface kinetics. *Atmos. Environ. A-Gen.* **27**, 2039-2050 (1993).
